# Thirteenth Annual Report of the Secretary of the State Board of Health of Michigan

**Published:** 1887-04

**Authors:** 


Thirteenth Annual Report of the Secretary of the State Board of Health of the State of Michigan, *for the year ending September, 1885; pp. xxxviii and 293.*

In this volume are included the reports by Dr. George M. Sternberg, United States Army, and Professor V. C. Vaughan, upon the poisonous cheese which caused so much sickness in Michigan during the year from August, 1883, to August, 1884, a full account of the history of which is given in the previous report of the State Board of Health. Dr. Sternberg thinks that the poisonous quality in the cheese is due to ptomaines, possibly developed by the presence of a non-pathogenetic micrococcus, which he found in the samples furnished him.

Professor Vaughan found a substance which forms in needle-shaped crystals, and for which he proposes the name tyrotoxicon. This substance produces symptoms which closely resemble those caused by the poisonous cheese, and Professor Vaughan thinks it is "not of bacterial origin."

This volume is largely given up to meteorological conditions, and communicable diseases in Michigan during the year. E. W.

